# Multispectral Satellite Image Analysis for Computing Vegetation Indices by R in the Khartoum Region of Sudan, Northeast Africa

**DOI:** 10.3390/jimaging9050098

**Published:** 2023-05-11

**Authors:** Polina Lemenkova, Olivier Debeir

**Affiliations:** Laboratory of Image Synthesis and Analysis (LISA), École Polytechnique de Bruxelles (Brussels Faculty of Engineering), Université Libre de Bruxelles (ULB), Building L, Campus du Solbosch, ULB—LISA CP165/57, Avenue Franklin D. Roosevelt 50, 1050 Brussels, Belgium; olivier.debeir@ulb.be

**Keywords:** remote sensing, Sudan, Khartoum, Nile river, image analysis, image processing, information retrieval, programming, Africa, computer vision, 91.10.Da, 91.10.Jf, 91.10.Sp, 91.10.Xa, 96.25.Vt, 91.10.Fc, 95.40.+s, 95.75.Qr, 95.75.Rs, 42.68.Wt, 86A30, 86-08, 86A99, 86A04, Y91, Q20, Q24, Q23, Q3, Q01, R11, O44, O13, Q5, Q51, Q55, N57, C6, C61

## Abstract

Desertification is one of the most destructive climate-related issues in the Sudan–Sahel region of Africa. As the assessment of desertification is possible by satellite image analysis using vegetation indices (VIs), this study reports on the technical advantages and capabilities of scripting the ‘raster’ and ‘terra’ R-language packages for computing the VIs. The test area which was considered includes the region of the confluence between the Blue and White Niles in Khartoum, southern Sudan, northeast Africa and the Landsat 8–9 OLI/TIRS images taken for the years 2013, 2018 and 2022, which were chosen as test datasets. The VIs used here are robust indicators of plant greenness, and combined with vegetation coverage, are essential parameters for environmental analytics. Five VIs were calculated to compare both the status and dynamics of vegetation through the differences between the images collected within the nine-year span. Using scripts for computing and visualising the VIs over Sudan demonstrates previously unreported patterns of vegetation to reveal climate–vegetation relationships. The ability of the R packages ‘raster’ and ‘terra’ to process spatial data was enhanced through scripting to automate image analysis and mapping, and choosing Sudan for the case study enables us to present new perspectives for image processing.

## 1. Introduction

### 1.1. Background

In this paper, we introduce the application of R libraries for satellite image processing in the context of computing vegetation indices (VIs) from the multispectral bands. Furthermore, we focus on calculating several satellite-derived VIs by discussing their technical details and differences. The test dataset includes the Landsat 8–9 OLI/TIRS images collected on Khartoum city around the confluence between the White and Blue Niles. The images were used to analyse the changes in the vegetation patterns and greenness through analysing the time series and visualising the dynamics in vegetation coverage. The standard methods for such tasks are based on using the traditional Geographic Information System (GIS) software [1] and involve many routine operations [2,3,4,5]. Instead of using such conventional approaches, we apply several libraries of R such as ‘raster’ and ‘terra’ and auxiliary packages in a principled way designed to obtain a better workflow for the image analysis involved.

The satellite sensors observing the Earth provide remote sensing data which allow one to highlight the links between the hydrological and climate processes leading to desertification and vegetation responses in environmental studies. The importance of the use of remote sensing data and advanced methods of image processing was raised in earlier studies [6,7,8,9]. Satellite images can be used to analyse the links between complex hydrological and climate processes and vegetation responses that lead to desertification. For example, Landsat images are known to be a reliable source of data for relatively accurate techniques for classifying time-series and detecting forest and land cover types [10,11,12,13], computing vegetation indices [14,15,16] and specifically desertification [17] to show the advance or retreat of arid areas using the analysis of satellite images. Specifically, for the Sudan and Nile Basin, the Landsat data are a precious source of information due to data scarcity [18] regarding regular measurements of rainfall, streams run-off and weather stations data. This highlights the importance of the remote sensing data for climate-related studies of Sudan.

GIS was one of the earliest computer-based applications for satellite image processing developed for the empirical investigations of the environmental parameters derived from remote sensing data. Since the 1990s, researchers were able to accurately compute the vegetation indices derived from the images using GIS. Previous studies evaluated the performance of the Earth Resources Data Analysis System (ERDAS) Imagine in computing vegetation indices to assess desertification in semi-arid regions, emphasising that the Normalized Difference Vegetation Index (NDVI) classification is suitable for assessing land degradation using the NDVI pixel range [19]. Others examined the links between the NDVI and the salinity characteristics in the surface water, groundwater and soils using the ArcGIS [20]. Furthermore, Ezaidi et al. applied ENvironment for Visualizing Images (ENVI) GIS for computing the NDVI for a time series of the Landsat images to assess the degradation in vegetation [21]. All these studies illustrate the traditional approaches for computing vegetation indices by GIS.

As a general overview, GIS enables to calculate vegetation indices through the manipulation of Landsat images using map algebra. However, the efficacy of the computational approaches must be questioned, as more advanced tools of spatial data analysis enable the application of modelling methods for computing the VIs. Over the course of the past 20 years, there has been significant progress in programming applications in environmental studies towards automation methods in image processing, such as R. Programming methods in geoinformatics have undergone significant evolution in recent decades, and now they generally result in higher accuracy, and diverse computational approaches in a variety of libraries and pre-programmed packages for data processing.

For instance, ref. [22] demonstrated the use of generalised additive models to study the relationships between vegetation types and NDVI. Furthermore, ref. [23] demonstrated the use of Holt-Winters modelling to detect trend and seasonal vegetation patterns using Python’s Scikit-Learn library, and [24] demonstrated the use of the partial least squared regression (PLSR) model in the SIMCA-P software to track the non-linear relationships between the NDVI and climatic variables. The recent development of machine learning tools through the use of the Python programming language enabled the use of a convolutional neural network (CNN) and deep neural networks (DNNs) for the environmental analysis and estimation of the VIs [25,26,27,28]. Such technical advances are beneficial for image processing and spatial data analysis compared to traditional GIS methods [29,30,31,32,33,34]. Considering such advantages of the programming languages in the automation of geospatial data analysis, the algorithms of R present a faster and more effective approach to implement image processing scripts.

Compared to the aforementioned programming approaches, the use of R in the context of computing VIs presents a more straightforward approach. Similarly to GIS approaches, R is adjusted to the processing of the Landsat bands; at the same time, the flexibility of R being a programming language allows the full control of the data processing and a high level of automation. Examples of R used for computing VIs include the ‘raster’ package for computing the NDVI [35], the ‘npphen’ R package to assess the NDVI anomalies and evaluating spatio-temporal changes [36], the ‘akima’ R package to interpolate environmental parameters [37] or the ‘RStoolbox’ package for computing the NDVI [38]. Such methods are comparable to our approach; however, they are largely tied to the formulation of a specific problem applied to the target areas. To the best of our knowledge, no previous studies reported on the use of R for computing the VIs in Sudan. This study fills this gap and highlights the benefits of using advanced techniques to compute the VIs specifically for the Sahelian landscapes of southern Sudan, which are prone to desertification.

### 1.2. Objectives and Motivation

The main purpose of the current paper was to apply an R-based method of satellite image processing that can extract information on several vegetation indices for evaluating the vegetation patterns in the arid environmental setting of southern Sudan. The method of R utilises the concept of scripting libraries and suggests a way to obtain values of spectral bands for computing diverse vegetation indices that describe the vegetation greenness and health. Subsequently, this builds an approach to analyse the remote sensing data associated with a spectral signature of land surface features visible in the spaceborne data. The resulting series of the computed vegetation indices can be evaluated to support environmental monitoring and mapping in semi-arid and arid areas of eastern Africa.

In this paper, we address the issue of using the R programming language in geospatial studies with a particular case of satellite image processing. Compared with the existing literature, the main contributions of this paper are highlighted as follows:

- To the best of our knowledge, this is the first paper in the literature that focuses on the application of R libraries for computing several vegetation indices over the area of Khartoum, Sudan;
- As opposed to previous studies, we present the application of ‘raster’ and ‘terra’ packages of R for remote sensing data analysis instead of the traditional GIS software;
- We further extend the use of Landsat 8–9 OLI/TIRS sensors to extract geospatial information from multispectral images;
- We comprehensively evaluate the performance of different vegetation indices for the case of semi-arid vegetation patterns in southern Sudan. To this end, we apply the R algorithms to calculate five different VI which differ in computational approaches and tuned to diverse environmental aspects of vegetation: (1) Normalised Difference Vegetation Index (NDVI); (2) Normalised Difference Water Index (NDWI); (3) Infrared Percentage Vegetation Index (IPVI); (4) Optimised Soil-Adjusted Vegetation Index (OSAVI); and (5) Green Normalised Difference Vegetation Index (GNDVI);
- We provide an overview of the major environmental aspects of Sudan which include recent issues of desertification related to semi-arid climate, land cover changes and hydrology of the Nile which controls the cycle of vegetation growth in Sudan.
- A summary of R scripts used for computing and mapping vegetation indices is reported, to provide the reader with the technical reference of the applied methods.

The remainder of this paper is organised as follows. We review the environmental setting of the study area with related works in the following subsection. In Section 2, Materials and Methods, we provide a description of the data and methods used in this study. We describe the data used and report their major characteristics in Section 2.1, Data. We introduce an R language concept, present a principle of R-base libraries for computing vegetation indices and propose the use of ‘raster’ and ‘terra’ libraries in Section 2.2, Methodology. In Section 2.3, Calculating Vegetation Indices, we provide a comprehensive experimental evaluation computing several vegetation indices, providing details on the algorithms, the differences in their implementation and target applications. In Section 3, Results, we report on the results of five computed vegetation indices and compare their performances on the vegetation of southern Sudan. We conclude this paper with a discussion in Section 4 and provide closing remarks in Section 5. The main metadata of the satellite images and programming listings of the R scripts are provided in the Appendix A and Appendix B.

### 1.3. Study Area

**Figure 1:**
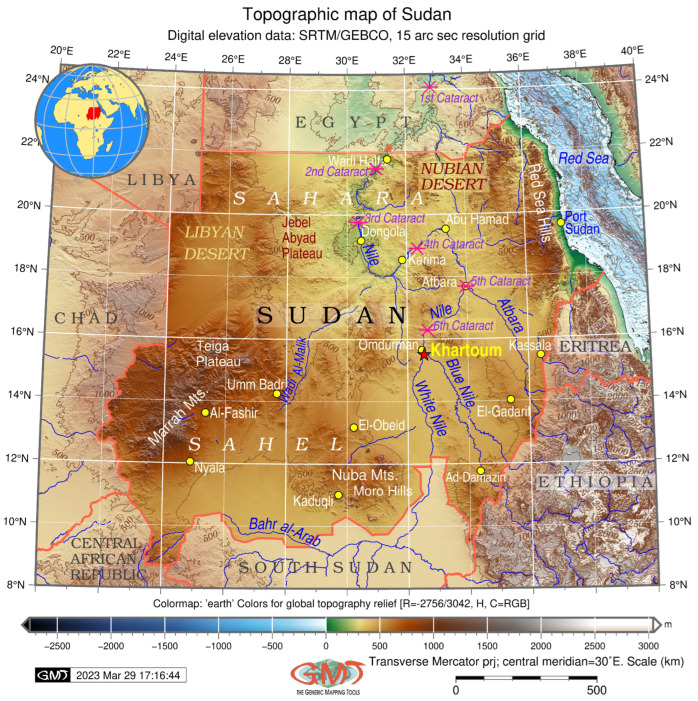
Topographic map of Sudan. Mapping software: Generic Mapping Tools (GMT) scripting toolset version 6.1.1, [51]. Data source: GEBCO/SRTM. Cartography source: authors.

Desertification is one of the most destructive climate-related issues in Sudan, which is located southwards of the Sahara Desert in the Sudan–Sahel region of Africa, (Figure 1). The desertification results from various factors including climate change and human activities. The climate drivers include rising temperatures, decreased relative humidity [39], increased soil salinization [40], seasonality and a decline in the rainfall [41], instability and irregularity of wet and dry seasons [42], variability of precipitation [43,44] and overall water deficit [45]. Recent studies such as [46] reported frequent and severe drought periods in recent decades resulting in several cycles of famine in Sudan affecting farmers and local populations. For instance, chronic drought was reported in the Sahel region of Darfur [47], and droughts have been reported in central Sudan [48] and eastern Sudan [49,50].

Other factors include changed patterns of cloudiness and atmospheric radiation, resulting in high evapotranspiration in the extreme north near the Sahara, southern Sahel and the savannah, affecting the vegetation growth [52]. Hence, the environmental challenges in the semi-arid climate of Sudan affect vegetation patterns which are controlled by the regional climate setting and variations in rainfall, evaporation, cloudiness, atmospheric radiation and other climate-related processes. Recent droughts and decreased rainfall affect vegetation growth and health due to the lack of humidity in the air. As a consequence, this initiated gradual processes of desertification in Saharan-Sahelian Africa, with the expansion of drylands and the degradation of the landscapes.

These processes trigger environmental degradation, especially in vulnerable zones of central and southern Sahelian zone of Sudan [53,54] and the savannah [55]. The most endangered region of Sudan prone to the risks of desertification lies between 12° and 18° N, near its capital—Khartoum. The flat topography of the country exacerbates the desertification problems and increases the effects of dust and sand storms that often occur in the northern regions of Sudan [56,57,58,59]. Specifically, this includes the effects of the creeping sands and dunes from the areas of Sahara, Nubian and Libyan deserts, as can be seen in Figure 1. The consequence of this is deforestation, soil desiccation, changed land cover types [60] and decreased biodiversity. The latter includes the extinction of wild species in oases, and consequences of land cover changes include the imbalance of ecosystems, spread of invasive species [61] and disturbance of natural habitat patterns [62].

**Figure 2:**
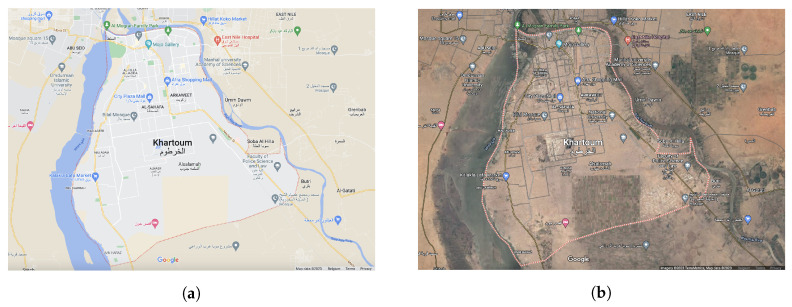
The confluence of the White Nile and Blue Nile in Khartoum, visualised based on the map and aerial images from Google Earth: (**a**) Google Earth Map; and (**b**) Google Earth Image. The non-English characters in the map present the names of the topographic objects added automatically on the Google Map and Google Earth data using national official language of the country. Here the Arabic translations are added for all the settlements and objects represented on a map of Sudan on the Google Map and Google Earth data.

Out of the territory of Sudan, 70% belongs to the Nile basin [63], with five out of the six Cataracts of the Nile located within Sudan (Figure 1). Moreover, roughly 2/3 of the territory of the Nile lies within Sudan with its major tributaries—the While Nile and Blue Nile—joining near Khartoum [64]. Hence, the specifics of the Khartoum region are tightly connected to the Nile river system and the confluence of the White Nile and Blue Nile, as shown in Figure 2. The impact that the Nile river has on the environment includes occasional floods, soil erosion and the succession of vegetation on the islands and banks [65], the accumulation of sedimentation [66], or the growth in aquatic weeds [67]. While providing a specific place for a wetland habitat of rare species, the Nile river is also an essential water artery of the country and plays multiple crucial roles. It is a source of hydropower in Sudan [68], an important transport waterway and provides water resources for 85% of the population and irrigated agriculture [69]. Moreover, the functionality and structure of the riparian vegetation in Sudan strongly depend upon the hydrology of the Nile, especially in its southern region near the confluence of the White Nile and the Blue Nile.

The social factors of desertification include processes related to land management and agriculture policymaking [70]. Although agriculture and livestock play an important role in the economy of Sudan and constitute major sources of livelihood [71], unsustainable agricultural expansion and farming contribute to Sudan’s desertification [72] since uncontrolled agriculture results in soil erosion, compaction and nutrient depletion, changed irrigation patterns [73] and the increased use of pesticides [74] which contribute to land cover changes. Moreover, cattle overgrazing also modifies the landscape structure and contributes to desertification [75,76,77]. The intense practice of urban agriculture in Khartoum aims to meet the increasing food demands of the growing population [78] which results in additional pressure on the environment on the one hand, and a gradual increase in the built-up area on the other. The confluence of the Blue Nile and White Nile forming its major artery of the Greater Nile near Khartoum results in a high concentration of the population in this district. Furthermore, consequences of the desertification also drive people into Khartoum, thereby increasing the urbanisation and the environmental pressure in the region [79,80], and the Atbara River (Red Nile) has high levels of mining activities related to resource exploration [81].

Desertification leads to many negative environmental consequences such as land degradation in the arid and semi-arid regions of Sudan [82,83]. The social consequences of desertification in Sudan include increased droughts and associated famine due to affected food production and soil degradation in the Sahelian zone [84]. The environmental changes in the past highlighted links between the human occupation and responses from the environment [85]. For instance, the deep ploughing of the surface increased the susceptibility of soil to wind erosion, which leads to a severe decline in fertility and the formation of sand dunes [86]. Other examples of the effects of the desertification include reduced biological productivity [87,88] and a decreasing plant biomass, with an increase in the extremely dry desert areas. The cumulative effect of these factors include unpredictable and severe droughts, desiccation or aridification and dryland degradation or desertification in the dryland regions [89].

Climatic trends are reflected in the hydrology of the Nile, which shows high levels of variability in the river flow records [90] and variations in peak discharge [91]. For instance, the increasing trend in the natural water storage variation of the Nile can be detected in northern Sudan [92]. Such fluctuations in the hydrology of the Nile necessarily affect the vegetation coverage in the banks and surrounding areas. Additionally, the patterns of the vegetation in the Khartoum Province are deeply connected to the regional geologic setting [93], the geomorphic structure and dominating soil types that control the distribution of the specific plants [94]. The White Nile is sensitive to rainfall and evaporation in lakes and swamps due to the specific hydrology of the region. Therefore, its response to occasional climatic events can be measured over long timescales [95] and the analysis of the vegetation patterns and soil erosion can be used for detecting vulnerable regions prone to desertification [96]. Nevertheless, despite efforts being put on water resources conservation and the rational exploitation of resources [97,98], environmental sustainability in the Sudanese region remains a challenge.

## 2. Materials and Methods

### 2.1. Data

**Figure 3:**
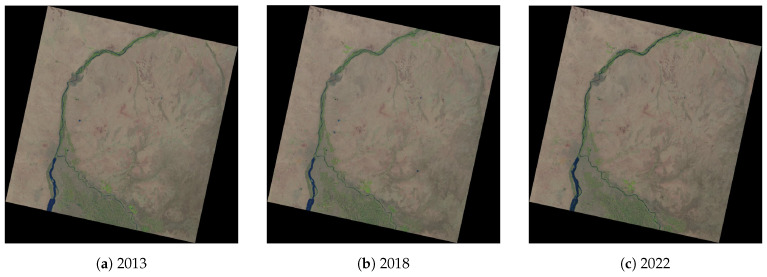
Landsat 8 OLI/TIRS image of White and Blue Niles in Khartoum, Sudan, in natural RGB colours in December: (**a**) 20 December 2013; (**b**) 18 December 2018; and (**c**) 21 December 2022.

We evaluate our approach on three datasets, with the Landsat 8 OLI/TIRS satellite images covering the region of southern Sudan on three different days, namely 20 December 2013, 18 December 2018 and 21 December 2022. The satellite images were selected and downloaded from the United States Geological Survey (USGS) EarthExplorer open repository for satellite images, aerial photographs and cartographic products (https://earthexplorer.usgs.gov/ (accessed on 13 February 2023)), Figure 3.

The choice of data is explained by the availability of the Landsat OLI/TIRS scenes, sensor spectral band pass, the high spatial resolution of 30 m across the multispectral channels (1–7), the open availability of the cloud-free images (0% of cloudiness) and a regular survey which provides the comparable gap between the target years (2013 and 2022) when the images were collected for the analysis of changes in vegetation (Figure 3). The information on the cloudiness of the scenes was extracted, among all other technical parameters, from the .xml file of the Landsat OLI/TIRS scenes where all the metadata are listed. The most important metadata are listed in the Appendix A, Table A1. The metadata on the Landsat OLI/TIRS satellite images were used in this study.

Among the available Landsat products, the Landsat OLI/TIRS sensor was selected, which has better technical and spectral characteristics compared to older sensors. Thus, compared to the Landsat-7 ETM+, the Landsat 8–9 OLI/TIRS has narrower spectral bands, higher calibration, finer radiometric resolution and geometry and better signal-to-noise parameters [99]. This results in a more narrow and precise bandwidth in red and NIR channels which differ from older sensors. For instance, the Landsat 8–9 OLI/TIRS sensor has 0.64–0.67 µm in the Red band (Band 4) and 0.85–0.88 µm in the NIR (Band 5), while the Landsat 7 ETM+ sensor has a coarser range of 0.63–0.69 µm in the Red (Band 3) and 0.77–0.90 µm in NIR (Band 4). The spectral characteristics of the older Landsat products (Landsat 4-5 TM and Landsat 1-5 MSS) has even coarser parameters. Since the Landsat 8 OLI/TIRS was launched on 11 February 2013, there are no earlier images, and the earliest possible date for the cloud-free image in the target month of December was 20 December 2013.

The images were taken within the 5-year gap between December 2013 and December 2018 and the four-year gap between December 2018 and December 2022, which gives the comparable intervals of the periods between the scenes. Examples of similar studies that also use few satellite images for the analysis of land cover changes or environmental monitoring include the use of the Landsat 5-TM, 7-ETM+, 8-OLI or Sentinel-2 scenes [100,101,102,103,104,105]. Using images over a more frequent period (e.g., every two months) throughout each year is biased by the natural growth cycles of plants: the periods of seedling formation, the development of sprouts, differentiation of leaves and the growth of plants from small to adult stages. Therefore, it is important to observe the difference in vegetation indices between the images taken in a particular month, which remains the same for all the images taken on different years. Hence, in our case, all the images were collected in December but in different years: 2013, 2018 and 2022.

The selected Landsat images were processed with the aim of computing vegetation indices using the algorithms of R to enable the analysis of the dynamics of vegetation over this period. Using a large dataset (e.g., dozens of scenes) allows detailed environmental investigation. Nevertheless, this creates technical challenges in terms of processing and organising data, which is possible in studies focused on big data processing, where multiple images are analysed as time series using special approaches [106,107,108]. Within the scope of this study, we used three Landsat OLI/TIRS images with the aim of investigating the spatio-temporal aspects and trends of short-time variations in vegetation patterns in southern Sudan during nine years. The images were collected in December in the years 2013, 2018 and 2022, with a gap enabling the detection of minor changes. Such a time span effectively represents the changes in the vegetation patterns in a given month of the observed years in southern Sudan.

The results of spatial analysis were applied to investigate the changes in vegetation patterns in the Khartoum region using vegetation indices computed during the period of nine years for each Landsat scene. The summary of the data is presented in Table 1. All images have 0% cloudiness and Path/Row ID 173/49. The sensor ID is common for all scenes: Landsat 8–9 OLI/TIRS (Operational Land Imager and Thermal Infrared Sensor), Collection 2 Level-2. Image source: the USGS [109]. The major technical and geodetic metadata common for all the images are the following: Datum and Ellipsoid WGS84; Product Map Projection L1—Universal Transverse Mercator (UTM); UTM Zone 36; Sensor ID OLI TIRS; Landsat Processing Software Version LPGS_16.2.0. The full list of metadata is provided in Table A1.

### 2.2. Methodology

With this study, we process the satellite images using R version 4.2.2 (R Core Team, 2020) and its packages for the computation of VIs and spatial data analysis [110]. The extraction of VIs for the analyses of vegetation health by traditional software, which is conceptually straightforward due to raster algebra, may nevertheless lead to a consequent computing time. The VIs are used as robust and reliable indicators of plant health and greenness because they indicate at the level of leaf chlorophyll in plants. As such, the VIs reflect the functional features of canopy and the parameters of vegetation coverage essential for environmental and climate analytics.

In this study, we compute the VIs and analyse the maps based on these indices for the years between 2013 and 2022. In a first step, the necessary libraries ‘terra’ version 1.7-18, ‘RColorBrewer’ version 1.1-3 [111], ‘Hmisc’ version 5.0-1 [112] and ‘pals’ version 1.7 [113] were activated and a working folder was defined. The ‘terra’ library is the key package which was used for raster algebra and calculation of the VIs, while the other libraries were used as auxiliary packages for the graphical refinements and proper visualisation of the plots. Then, we defined separate functions for each of the VIs using their relevant formulae. Finally, we uploaded the necessary Landsat bands in the active folder and performed the calculation of each of the VIs by R scripts, as presented below for each corresponding index.

**Figure 4:**
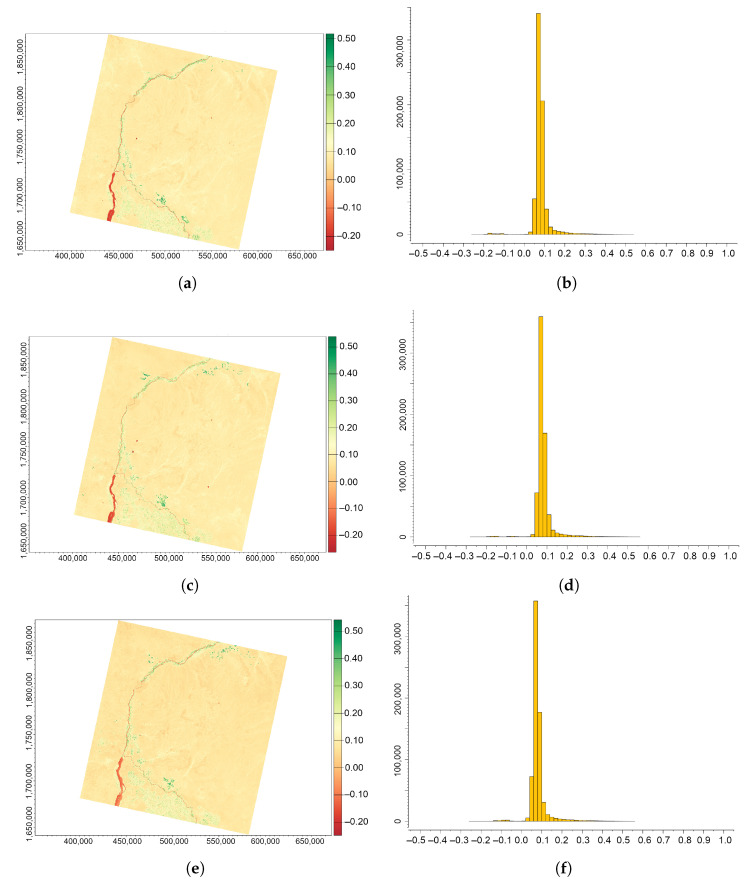
NDVI computed from the Landsat 8 OLI/TIRS images of the confluence between the White Nile and Blue Nile, Sudan, for three years (always in December): (**a**) 2013; (**b**) 2018; (**c**) 2022; (**d**) histogram for 2013; (**e**) histogram for 2018; and (**f**) histogram for 2022.

**Figure 5:**
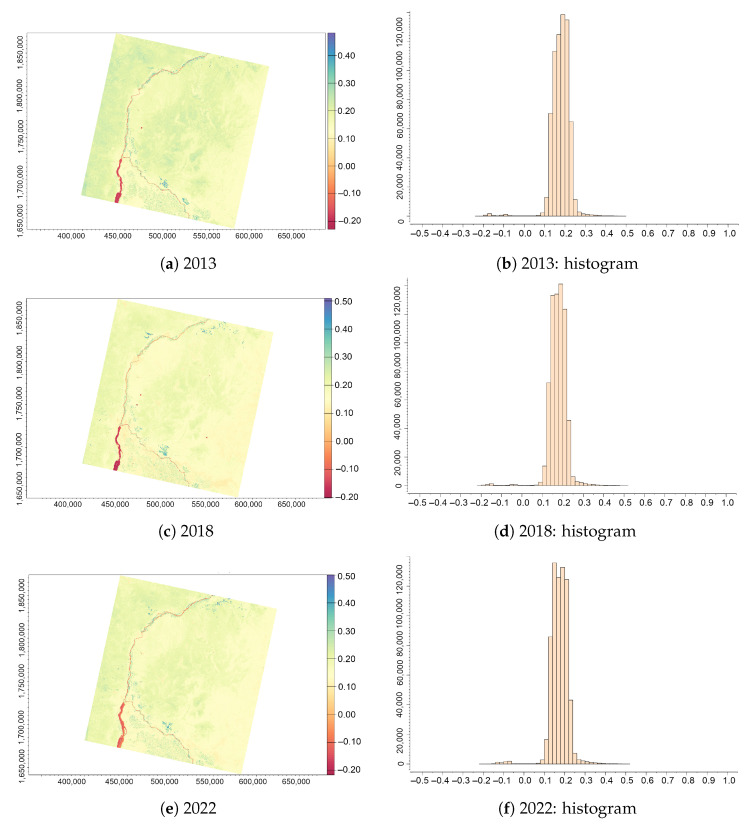
GNDVI computed from the Landsat 8 OLI/TIRS images of the confluence between the White Nile and Blue Nile, Sudan, for three years (always in December): (**a**) 2013; (**b**) 2018; (**c**) 2022; (**d**) histogram for 2013; (**e**) histogram for 2018; and (**f**) histogram for 2022.

**Figure 6:**
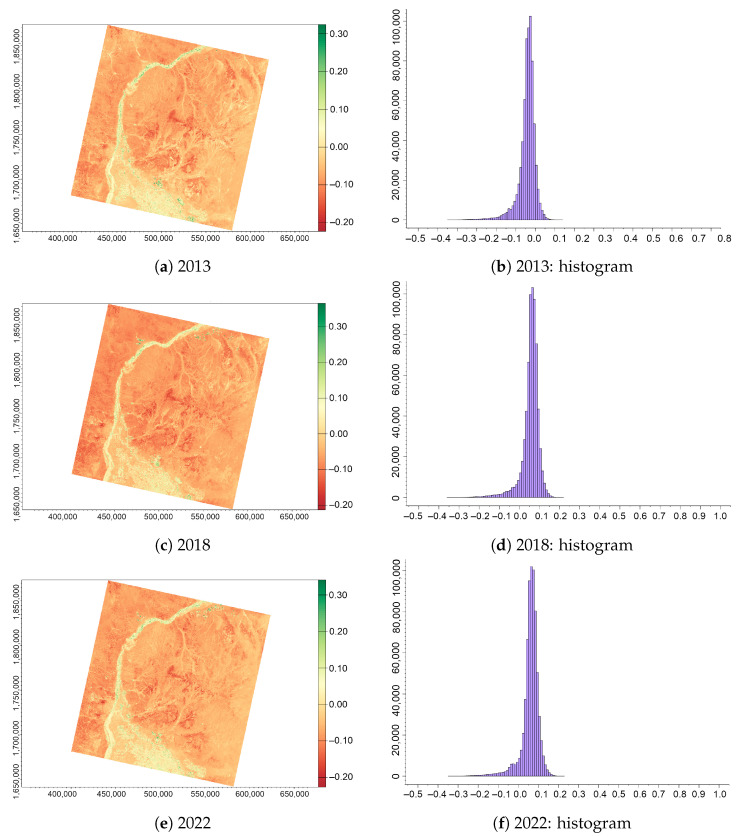
NDWI computed from the Landsat 8 OLI/TIRS images of the confluence between the White Nile and Blue Nile, Sudan, for three years (always in December): (**a**) 2013; (**b**) 2018; (**c**) 2022; (**d**) histogram for 2013; (**e**) histogram for 2018; and (**f**) histogram for 2022.

**Figure 7:**
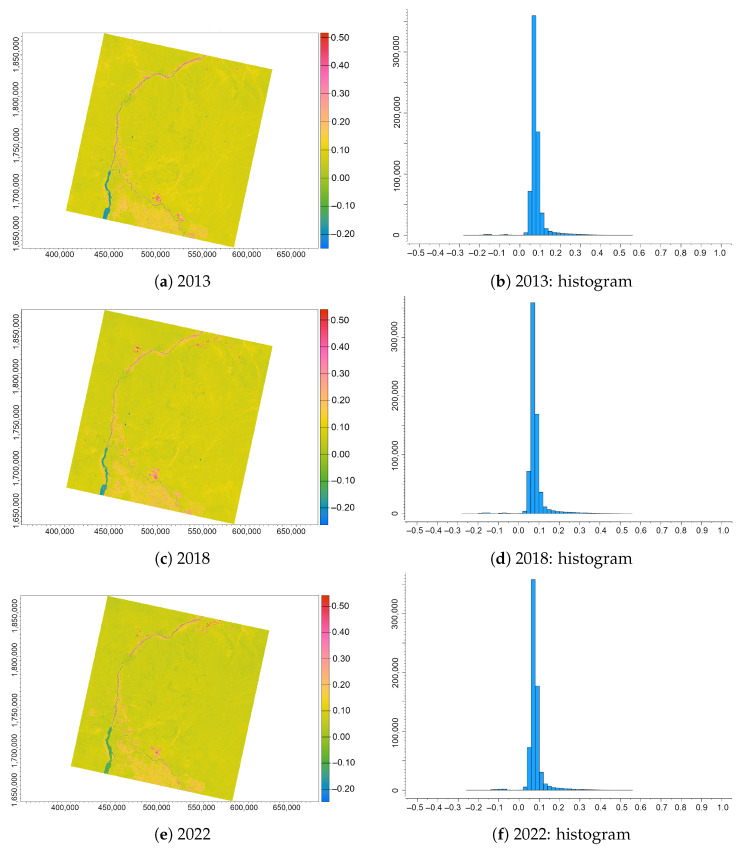
OSAVI computed from the Landsat 8 OLI/TIRS images of the confluence between the White Nile and Blue Nile, Sudan, for three years (always in December): (**a**) 2013; (**b**) 2018; (**c**) 2022; (**d**) histogram for 2013; (**e**) histogram for 2018; and (**f**) histogram for 2022.

**Figure 8:**
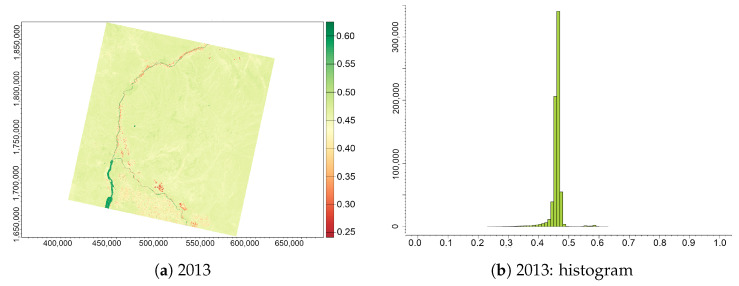

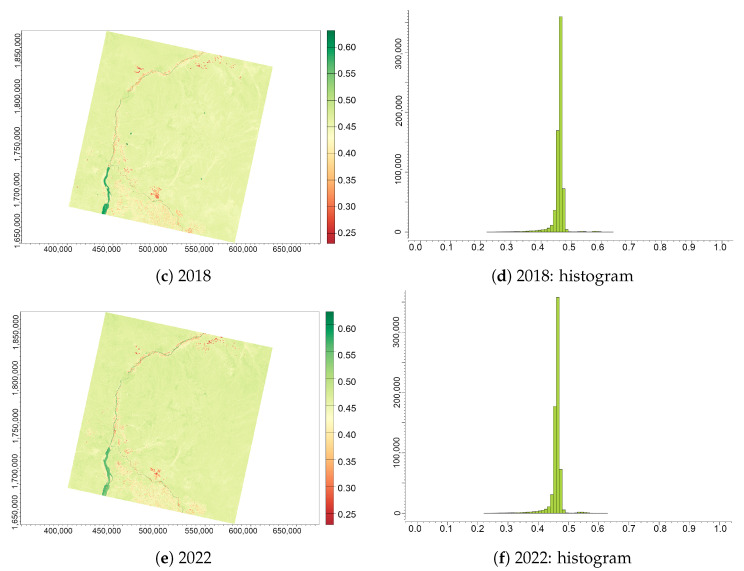
IPVI computed from the Landsat 8 OLI/TIRS images of the confluence between the White Nile and Blue Nile, Sudan, for three years (always December): (**a**) 2013; (**b**) 2018; (**c**) 2022; (**d**) histogram for 2013; (**e**) histogram for 2018; and (**f**) histogram for 2022.

To compute the VIs, we used the ‘terra’ R package approach which allows the assessment of the vegetation patterns using computational formulae to study vegetation greenness and to assess the increase in desert areas. There are two important advantages of the R approach with regard to the estimation of vegetation health using histograms showing a data distribution for the analysis of the phenological changes in canopies: (i) the embedded map algebra is adjusted to describe any type of VI using the function (vi<−function(img,k,i)) (see the R scripts in Appendix B) which enables one to vary Landsat bands for computational purposes in different formulae; and (ii) it calculates the data frequency distribution showing the number of pixels that correspond to higher greenness, from which the trends to desertification can be assessed using image analysis for various years. The full scripts used for plotting the Figure 1, Figure 4, Figure 5, Figure 6, Figure 7 and Figure 8 are provided in the GitHub repository: https://github.com/paulinelemenkova/Mapping_Sudan_Scripts (accessed on 23 February 2023).

### 2.3. Calculating Vegetation Indices

#### 2.3.1. Normalised Difference Vegetation Index (NDVI)

The Normalised Difference Vegetation Index (NDVI) [114] is calculated as a ratio between the sum and the difference between the two Landsat bands—near-infrared (NIR) and red. The NDVI is the most well known and widely used ecological indicator for assessing vegetation conditions due to the optimally created formula that includes the spectral properties of red and NIR bands. Thus, compared to the other bands, the content of the chlorophyll in healthy vegetation is much more significantly reflected by the NIR band. At the same time, it absorbs red light. Therefore, using these two bands in a well-established combination gives robust results indicating the presence and amount of chlorophyll in leaves. Such effectiveness of the NDVI resulted in its numerous applications to the environmental analysis [115,116,117,118]. The NDVI is computed using Equation (1):

(1) $$\mathrm{NDVI}=\frac{\left(\mathrm{NIR}-\mathrm{Red}\right)}{\left(\mathrm{NIR}+\mathrm{Red}\right)}$$

Using R, we computed the NDVI using a script presented in Appendix B, Listing A1.

#### 2.3.2. Green Normalised Difference Vegetation Index (GNDVI)

The GNDVI index is an updated and modified NDVI to remotely sense the presence and vitality of vegetation. Compared to the NDVI, it also uses the NIR Band for computation but replaces the Red Band (i.e., Band 4 for the Landsat 8–9 OLI/TIRS with a spectral band between 640 and 670 nm wavelengths) by the Green Band (Band 3 for the Landsat 8–9 OLI/TIRS with a spectral band between 530 and 590 nm wavelengths) from the visible spectrum. Therefore, the GNDVI estimates the chlorophyll content more precisely compared to the NDVI and enables the analysis of the photosynthetic activity in plants. The applications of the GNDVI are mostly intended at detecting wilted, ill or aging plants and monitoring plant stress.

Moreover, it is also suitable for estimating the nitrogen content in the plant leaves, as well as monitoring mature vegetation coverage in the final stages of the crop cycle or vegetation with dense canopy. Similarly to the NDVI, the range of values in the GNDVI also varies between −1 and 1, where negative values are associated with the areas of water or bare soil, while higher values indicate healthy vegetation. In this study, the GNDVI was computed due to its technical and practical advantages. Thus, its advantages include the low number of required spectral bands (only NIR and Green, as indicated in the formula of Equation (2)) which results in easy computation in R, and reliability in the analysis of vegetation health due to the more precise estimation of the chlorophyll content compared to the NDVI. The formula for the GNDVI used for computation is given in Equation (2):

(2) $$\mathrm{GNDVI}=\frac{\left(\mathrm{NIR}-\mathrm{Green}\right)}{\left(\mathrm{NIR}+\mathrm{Green}\right)}$$

For the Landsat 8–9 OLI/TIRS bands, the Green channel corresponds to the Band 3 and Red channel—to Band 4. The adjustment to the GNDVI is based on the maximum sensitivity of the reflectance of leaves since the reflectance near 670 nm of the Red Band is not sensitive to the chlorophyll concentration due to the saturation of the relationship between absorption and chlorophyll concentration [119]. Therefore, the replacement of the Red Band by the Green Band results in a more accurate estimate of the concentration of chlorophyll and the rate of photosynthesis. Using R syntax, we computed the GNDVI using the script presented in Appendix B and Listing A2.

#### 2.3.3. Normalised Difference Water Index (NDWI)

Developed by Gao in 1996 [120] as a complementary to the NDVI, the NDWI is strongly related to the plant water content as it is sensitive to the liquid water content of leaves based on the ratio between the difference and sum of NIR and SWIR. The second version of the NDWI developed by [121] uses a Green band instead of SWIR which can be used to detect the surface open water areas as well as evaluate the level of turbidity. In this case, the NDWI is computed using the ratio between the difference and sum of the NIR and visible Green Bands [122]. In our study, we used the first version developed by Gao following Equation (3):

(3) $$\mathrm{NDWI}=\frac{\left(\mathrm{NIR}-\mathrm{SWIR}\right)}{\left(\mathrm{NIR}+\mathrm{SWIR}\right)}$$

The advantages of the NDWI include that it lessens the effects from the reflectance of soil and vegetation foliage which makes it suitable for sandy areas such as in Sudenese Sahel. A special value of the NDWI for environmental monitoring is that it can be used for indicating areas with water deficit [123]. Therefore, the NDWI presents a useful and precise tool in the assessment of the health of vegetation in arid areas prone to droughts, such as Sudanese Sahel, through computing the moisture level in plants as the detected water content in leaves. Thus, during the drought periods, foliage is strongly affected by the water deficit which results in plant illness. Specifically for the agricultural areas of Khartoum, this leads to the crop failure and decreases the production which affects the harvest. Detecting the plant water stress is essential to evaluate the current state of plants and make a prognosis for future development, e.g., by indicating the areas that are in need of irrigation. Using the R library ‘terra’, we computed the NDWI using the script presented in the Appendix B in Listing A3.

#### 2.3.4. Optimised Soil-Adjusted Vegetation Index (OSAVI)

Originally developed by [124], the Optimised Soil-Adjusted Vegetation Index (OSAVI) is well adjusted to monitor vegetation health, specifically in regions with low vegetation coverage, such as the Sahelian region of Sudan, due to corrections of bare soil and desert areas. Specifically, the OSAVI uses a defined value of 0.16 as a factor to adjust the canopy background, as this value gives higher soil variation compared to the original SAVI. At the same time, the OSAVI has a higher sensitivity to vegetation coverage that covers more than a half of the area in percentage. Due to such characteristics, the OSAVI is suitable for monitoring the existing productivity changes in agricultural areas near Khartoum and along the banks of the Nile river. Moreover, the corrections of the reflectance from bare soil and lands enables the detection of areas prone to droughts using the comparison of the multi-temporal satellite images. Therefore, the application of the OSAVI is especially suitable in the regions with sparse vegetation, such as in southern Sudan, where soil is often visible through the canopy of vegetation. The OSAVI is computed using Equation (4):

(4) $$\mathrm{OSAVI}=\frac{\left(\mathrm{NIR}-\mathrm{Red}\right)}{\left(\mathrm{NIR}+\mathrm{Red}+0.16\right)}$$

In R language, we computed the OSAVI by the script shown in the Appendix B, Listing A4.

#### 2.3.5. Infrared Percentage Vegetation Index (IPVI)

The values of the Infrared Percentage Vegetation Index (IPVI) depend on the chlorophyll content in leaves. Developed originally by [125], it always has positive values ranging from 0 to +1 and indicates the photosynthetic activity of the canopy cover and the total chlorophyll content in the leaves. Furthermore, as a modified variation of the NDVI, it is especially suitable for indicating yellowish or shed leaves in the semi-arid areas such as southern Sudan, due to the adjusted spectral regions in the formula. Thus, the chlorophyll content directly depends on the nitrogen level in plants which contributes to the greenness of foliage and is reflected in the optical characteristics of leaves. In such a way, the IPVI is suitable for the periods of active vegetation development due to the indication of the leaf pigment content and variations showing always positive values above zero, in contrast with the NDVI. Therefore, the IPVI is useful to indicate the yield characterisation and stress level in leaves in the regions affected by droughts in the semi-arid areas to the north of Khartoum. In this way, the IPVI is a robust indicator of drought severity in southern Sudan and its effects on the arid and semi-arid ecosystems of Sudanese Sahel. Using the comparison of images covering the target test area on the given time periods in 2013, 2018 and 2022, the difference in the vegetation patterns shows the variations in the health conditions of leaves. The IPVI is computed using Equation (5):

(5) $$\mathrm{IPVI}=\frac{\mathrm{NIR}}{\mathrm{NIR}+\mathrm{Red}}$$

The computation of IPVI using the R library ‘terra’ was performed by the code presented in the Appendix B, Listing A5. The application of RStudio version 2023.03.0 + 386 [126] for image processing included the image processing, computing and plotting of the vegetation indices index using the Landsat 8 OLI/TIRS image.

## 3. Results

In order to evaluate the performance of our R approach using the ‘terra’ and ‘raster’ libraries, the experiments on the assessment of the time processing were conducted; the first for an assessment of file processing and reading the data and the second for evaluating the graphical plotting and statistical analysis. While in practice, one would only detect the time of single image processing, which is important to evaluate the effect of the processing speed and the use of computer memory from the viewpoint of the effectiveness of R packages, which is an important characteristic when processing many images as time series data.

All the computations were performed using a computer MacBook Air with chip Apple M1 2020, operation system MacOS Ventura version 13.2.1, which uses 8 GB of superfast unified memory. On this machine, the technical advantages and capabilities of R and its geo-processing packages ‘terra’ and ‘raster’ were evaluated in terms of processing time. We quantified several computational aspects, which demonstrated the following results. 1. Time taken to read data: less than 1 s (i.e., the data were read instantly as soon as ‘enter’ was pressed); 2. Time taken to process data (s): 9 seconds to process the following commands corresponding to lines 7–20 in the case of Listing A1 R code for computing the NDVI: vi <- function(); filenames <- paste0(); landsat <- rast(); ndvi <- vi(landsat, 5, 4); options(scipen = 10,000); and colours <- brewer.rdylgn(100). Next, the graphical plotting of the image (lines 21–22 in example of the same Listing) was perform during 2 s, and the plotting of the histogram (lines 24–27) took 1 s. 4. The overall processing speed took seconds: 12 s for processing each Landsat OLI/TIRS image. 3. Memory utilisation (Mb) demonstrated effective parameters due to the Mac with chip Apple M1 is powerful in terms of RAM. Therefore, the highest memory consumption was 60 Mb for 15 maps which includes the computer 5 vegetation indices for 3 different calendar years.

The distribution of vegetation patterns in the Khartoum region varies in different years according to different vegetation indices. The statistics on these values are collected and summarised in Table 2 for a comparison of the variations in the VI values. Thus, we provide in Table 2 for each set of images their VI values for the years 2013, 2018 and 2022, derived from the R report on data analysis, as it appears in the output console. In the second to fourth columns, we indicate the years of observations and output results obtained by the R ’terra’ library algorithm. For each image, we collected the data’s minimal and maximal values to assess its global range. Thus, the results of the VI values derived from the Landsat 8 OI/TIRS images over the Khartoum region area are presented in Table 2. These data show the variations indicating changes in vegetation health. The maximal values indicate the healthy green vegetation which corresponds to greenness due to the chlorophyll content in the leaves. The changes between 2013 and 2018 show a decline in the values in the indices NDVI, GNDVI, NDWI and OSAVI, and a very slight increase in the IPVI. The analysis of data from 2018–2022 shows that there is a slight rise in values for the same indices expected for the IPVI, which demonstrates a slight decrease.

### 3.1. Normalised Difference Vegetation Index (NDVI)

The images of the NDVI (Figure 4) allow the differentiation of vegetation areas or cultivated areas from the soil in the region southwards from Khartoum and along the flow of the Greater Nile. The differentiation of the crops against other land cover types is possible due to the different values in the NDVI data range. Thus, the values of the NDVI vary within the range from −1 to +1 where negative values indicate water, lower values above 0 show bare soil and land, and mediocre values (around 1.30–0.50) signify sparsely vegetated areas. Higher values around +1 identify dense vegetation with a healthy canopy and green foliage. More specifically, crops thus have higher values compared to the bare land and cultivated areas. For instance, sparse vegetation ranges from 0.1 to 0.5, shrubs and meadows range from 0.2 to 0.3, dense green vegetation ranges over 0.6, cultivated areas have small values (less than 0.2) and empty areas of rocks or sand are between 0.1 and 0.2. Negative values indicate water. The low NDVI values were classified as non-vegetation (i.e., soil and bare land) and positive NDVI values were classified as vegetation.

The highest NDVI values (0.80–1.0) identify forest areas. For the desert areas dominated by sands such as Sudan, the NDVI values do not exceed 0.50. The minimal values determined according to the histograms obtained from the NDVI images for the years 2013, 2018 and 2022 were 0.27, 0.28 and 0.28 (Table 2), whereas the highest determined values were 0.72, 0.58 and 0.60 for the same years. The minimum and maximum values were derived from the computed vegetation indices to evaluate the data distribution extremes. These indicate the range in vegetation greenness where higher values represent healthy vegetation with dense canopy and green foliage while lower values represent bare land.

The pixels that were not classified as either soil, water or vegetation classes were identified as sand desert area (mostly beige-coloured in the images in Figure 4) pixels due to low NDVI values of approximately 0.2 or less. The lowest NDVI values worth less than 0.1 are classified as rocky surface and bare land.

The identification method of soil/water and vegetation is based on the characteristics of the NDVI which ranges from −1.0 to 1.0 with negative values indicating clouds and water, positive values near zero (<0.1) indicating bare soil, barren rock or sand in deserts. Shrubs, grasslands and other types of sparse vegetation have moderate NDVI values between 0.2 and 0.5. Higher positive values of NDVI correspond to dense green vegetation (>0.6 and <0.9) which correspond to crops at their highest growth stage. Broadleaf/boreal and tropical forests can also exhibit these values, but it is known that the area of Sudan does not have such vegetation types. The vegetation in Sudan includes sandy arid lands in the deserts, semi-deserts, savannah (grassland), croplands and inland floodplains along the Nile river, which made it easier for us to distinguish between different land surface types. A light ivory colour corresponds to the patterns of the dry creeks or beds of wadi ephemeral streams. The minimal and maximal values of the NDVI varying for the years 2013, 2018 and 2022 are as follows, from −0.2742168 to 0.7246868 in 2013, from −0.2817099 to 0.5796811 in 2018, and from −0.2791084 to 0.6044010 in 2022. As one can note, the maximal values decreased from 2013, which indicates the general trend in desertification; however, from 2018 to 2022, there is again a slight increase in the highest values which can be explained by increased precipitation and fluctuations in Nile hydrology.

### 3.2. Green Normalised Difference Vegetation Index (GNDVI)

The highest values of the GNDVI did not exceed 0.72 for the area of Sudan due to the scarce vegetation coverage and dominating desert areas, as shown in Figure 5. Negative values are associated with the areas of water or bare soil, while higher values indicate healthy vegetation and selected agricultural croplands along the banks of the White, Blue and Greater Niles. As seen from Figure 5, the GNDVI captured plant greenness with changes in vegetation health and greenness corresponding to the colour in the images varying from bright green to yellow on a sequence of images for 2013, 2018 and 2022. In this case, the variations in shadows of colours indicate the differences in plant vigour, which is a measure of the increase in plant growth or foliage volume through time after planting [127]. Moreover, this indicates the plant health which correlates with the irrigated lands with a higher level of moisture in the soil that can be distinguished against drylands and desert areas.

Further comparing the values in Table 2, the range of values for the GNDVI is narrower than that of the NDVI, with minimal −0.24, −0.28 and −0.23 for the years 2013, 2018 and 2022, while the highest values are 0.64, 0.57 and 0.72 for the same years. Accordingly, with greener plants visualised in the GNDVI, Figure 5 shows lower and better distinguished values. Moreover, the histograms present a more diversified range of values for various land classes: water, bare soil and sand, rocky areas of deserts and vegetation with agricultural areas and natural aquatic plants in the Nile river. Finally, the slight increase in the GNDVI values indicating photo synthetic activity in leaves is affected by changes in the water and N absorption in plants that includes aquatic vegetation in the bank areas of the White Nile.

### 3.3. Normalised Difference Water Index (NDWI)

The interpretation of the values from the resulting images based on the NDWI (Figure 6) is as follows: the negative values (from −1 to 0) signify areas with no vegetation or water content, while those above 0 show surfaces with a water content where the higher moisture level correlates with higher values of the NDWI, respectively. The NDWI values vary between −1 and +1, which depends on the total amount of water in leaves, the type of vegetation and the percentage of foliage coverage. Thus, sparse vegetation in semi-arid regions of the Sudanese Sahel will naturally have a lower value of the NDWI compared to the regions of dominating forests with dense canopy. The analysis of the NDWI images reveals contours of the dry creeks in the desert areas with stream patterns and beds of drainage courses that contribute to the differences in moisture content in soil (Figure 6).

Such patterns are indicative of moist wadi sediments and associated vegetation strips. The range of values in the NDWI shows slow dynamics in the lowest values that gradually increases from −0.40 in 2013 to −0.56 in 2018 and 0.57 in 2022, along with increasing highest values: 0.38 in 2013, 0.38 in 2018 and 0.42 in 2022. The values of the NDWI rise along with the increased leaf water content as well as the vegetation fraction cover. Therefore, higher values correspond to a higher water content in leaves and to an increase in canopy.

We suggest that the drivers are climate and hydrological factors such as higher precipitation levels or increased water discharge from the Nile, but we cannot support our surmise with numerical arguments and this pattern should be elucidated in future studies. Since the NDWI is specifically designed to monitor vegetation in drought-affected areas such as Sudan, such variations indicate increases in both the content of vegetation water because of the strong chlorophyll absorption in SWIR and the structure in plant canopy.

On the one hand, partial vegetation coverage in the desert areas of central Sudan results in soil effects on the NDWI values, especially for the landscapes with interspersed soils and vegetation patterns. Thus, when the leaf layer in the canopy decreases, the absolute values of reflectance in the spectral region of the NDWI are decreased, which results in lower values. The range of the NDWI values in Khartoum is narrow, which is seen on the histograms with most of the values varying from −0.20 to +0.20. More specifically, the interpretation of the values of the NDWI follows the ranges: positive values from +0.2 to +1.0—water area; from 0.0 to +0.2—flooded areas and high humidity; from −0.3 to 0.0: moderate drought soil and non-water surfaces; and from −1.0 to −0.3: drylands, drought and non-water surfaces. The NDWI indicates the moisture level of vegetation which is suitable for the sandy areas in the Sahelian Sudan.

### 3.4. Optimised Soil-Adjusted Vegetation Index (OSAVI)

The computed OSAVI is illustrated in Figure 7.

The OSAVI was the highest for 2013 with the maximal value of 0.72 (Figure 7), after which it dropped to 0.58 in 2018, indicating the decrease in the leaf chlorophyll content reflected in the OSAVI values; between 2018 and 2022, the values stabilised and slightly increased to 0.60 due to the effects of the White Nile hydrology and climatic variations.

Being a modified index derived from the NDVI, the OSAVI also has a range of values varying between −0.28 and +0.72, with negative values indicating the water areas of the White and Blue Niles, lower areas slightly above 0 and lower than 0.2 indicating bare land, sandy areas, soil with no or very sparse vegetation, while higher values close to +0.50 show a healthy vegetation and higher chlorophyll content in leaves. The OSAVI values typically range from −1 to +1 where high values indicate denser, healthier vegetation, lower values indicate less vigour and medium values correspond to the agricultural lands. The highest and minimum values in this region show the range in data which indicate the changes in the greenness of vegetation and the health of plant leaves.

The OSAVI image shows that the land classes with values between 0.30 and 0.40 representing sparse vegetation, 0.40–0.50 represent medium vegetated areas typically for agriculture lands and those above 0.50 represent occasional regions with highly vegetated area around the Blue Nile. The analysis of the OSAVI and comparison with other indices reveals that the OSAVI classifying pixels on the image was more detailed compared to the NDVI and GNDVI as related to resolution. Thus, it identifies vegetation better using spectral reflectance within each cluster of the data range which results in the detection of the biomass and leaf foliage areas in the regions covered by sandy soils and semi-arid lands such as Sudan.

### 3.5. Infrared Percentage Vegetation Index (IPVI)

The majority of values of the Infrared Percentage Vegetation Index (IPVI) for the area of Sudan, Figure 8, lies within a very narrow interval of 0.25–0.48, which matches the dominance of sandy dune areas and bare soils in the surrounding deserts. Note that all the values of IPVI are never negative and always positive due to the formula used in computation, which distinguishes this index from the previous ones.

Figure 8 indicates the results of the computed IPVI for the Khartoum region with the subplots (a,c,e) indicating changes in vegetative patterns related to desertification for the study area between 2013 and 2022, and the histograms in the subplots (b,d,f) indicating the related statistics obtained from R. The comparison of the vegetative cover and the expansion of sand coverage on the images produced by applying IPVI algorithms in R reveals that the extent of degraded land in the surroundings of Khartoum in southern Sudan has expanded from 2013 to 2022, as well as the overall effects of land degradation.

## 4. Discussion

The method of R ‘raster’ and ‘terra’ libraries is advantageous for satellite image processing aiming to compute vegetation indices. The computations were conducted on a MacBook Air PC (i.e., chip Apple M1 2020, MacOS Ventura v. 13.2.1, 8 GB of main memory). The evaluated processing speed reported in the Results section of this manuscript demonstrated technical advantages and benefits of R and its libraries ‘terra’ and ‘raster’. The performance of R libraries was evaluated in terms of a processing time with quantified computational aspects. The high speed of computations demonstrated by R is beneficial for the repetitive workflow of image processing when several scenes need to be processed sequentially for several years. Specifically, the high speed of reading the data and total time of image processing (12 s for each image) confirmed that R is advantageous for remote sensing data processing, computational and mapping tasks.

Moreover, using scripts enables to save time through the automation of repetitive tasks. This is more advantageous compared to the GIS with a traditional GUI interface used for satellite image processing, such as ENVI GIS, SAGA GIS or Erdas IMAGINE. This is especially useful for a computation of several images to calculate several VIs using embedded formulae. Furthermore, R scripts result in accurate machine-based graphics when processing the satellite images due to a high level of automation. Applying scripts customised for each scene increases the speed of data processing which is advantageous when working with several images. For instance, scripts call embedded functions to perform parts of computations and data processing. Moreover, scripts provide the quick plotting of maps and graphics that visualise the results. This helps to highlight the changes in vegetation patterns when maps visualise the VIs across several years for comparison.

A package-based approach of R is used in this paper as a target tool for remote sensing data processing which enables the computation of VI for the analysis of satellite images. The presented use of R libraries shows the usefulness of this approach as an analytical tool with which we computed several VI and thus revealed changes in the vegetation patterns of southern Sudan since 2013. The use of R presents opportunities for the sustainable environmental monitoring of Sudan through the integration of remotely sensed data and advanced programming technologies. In this paper, we proposed the application of R language for the computation of several vegetation indices using the ‘terra’ library and satellite images Landsat 8–9 OLI/ITIRS. Compared to traditional GIS approaches, the proposed method demonstrates high efficiency and effectiveness for geospatial data processing aimed at environmental analysis. In the performed experiments calculating VIs over the Landsat 8 OLI/TIRS data, our study has shown that, in most cases of the indices, a general trend in the decrease in VI values is notable for the data for 2013, 2018 and 2022, as shown in Table 2 and visualised in Figure 4, Figure 5, Figure 6, Figure 7 and Figure 8. This is clearly visible from the results obtained using the estimated parameters on indices NDVI, OSAVI and IPVI.

Using three satellite images that Landsat 8–9 OLI/ITIRS collected for the region of Khartoum area, we detected a slight decrease in vegetation greenness from 2013 to 2022 based on the comparison of the dynamics of the satellite-derived VIs. For each image, the computation of five of the presented VIs was performed for three corresponding years by applying our R scripts. The visualisation of these indices demonstrates a function of vegetation health, greenness and vigour in the conditions of the Sahelian climate of Sudan with variations over the analysed years. The proposed R implementation shows promising results when detecting anomalies in vegetation conditions in southern Sudan, as the VIs provide essential information on plant greenness reflected as chlorophyll content. The chlorophyll content relates to the water content in leaves which in turn is controlled by regional climatic settings and the hydrological processes of the Nile river and its tributaries—the White and Blue Niles.

We addressed the processing of satellite images by computing a series of vegetation indices for the regions prone to desertification in the Sahelian area of Sudan. Our approach represents each Landsat 8–9 OLI/TIRS image with five derived vegetation indices, each tuned for various environmental purposes, and compared them for the years 2013, 2018 and 2022: detecting dense vegetation with healthy canopy and green foliage (NDVI); estimating the canopy background in the regions with sparse vegetation and a low percentage of foliage coverage, which was typical for Sudan (OSAVI); evaluating the maturity of the canopy based on the leaf pigment content and nitrogen level in plants (IPVI); evaluating the concentration of chlorophyll the in leaves and the rate of photosynthesis (GNDVI); and indicating areas with water deficit (NDWI). As such, the comparative analysis of indices visualised for various periods can be used to identify the vegetation conditions during the environmental monitoring of the Sahel region of northeast Africa.

We presented further insights into raster image processing by R scripting and optimisation methods for a time series analysis of several scenes. The computational workflow demonstrated that the proposed algorithms of R perform far better in terms of the processing and analysis of spatial datasets. The experiments demonstrated that scientific scripting in the R language plays an important role in geospatial image processing, visualisation and analysis, since it enables the integration of various components or libraries including graphical packages used for mapping and plotting as well as packages for spatial data processing which can be integrated into a coherent scripting workflow. This study analysed three Landsat OLI/TIRS images which revealed the effective capabilities of R when testing the packages ‘terra’ and ‘raster’. However, the seasonal or interannual analysis should be based on a time series analysis that includes a more representative variety of datasets which is envisaged in future works as a continuation of this study.

## 5. Conclusions

In this paper, we highlighted that the performance of the R packages is effective for a framework of satellite image processing and enables the computation of vegetation indices. With the presented maps of the VIs made based on the Landsat OLI/TIRS images, we observed the changes in the vegetation patterns in the test area. We further showed that there is a trend to the growth and greenness of vegetation during the period of 2013–2022 in the study area of the confluence of the White and Blue Niles in southern Sudan, which is caused by the rising temperature and decreased precipitation due to the climatic and environmental impacts effects of desertification.

The Landsat 8–9 OLI/TIRS images contain a complex image texture with distinct values of pixels due to the different spectral reflectance of vegetation and other land cover types. Five VIs of vegetation distribution analysis have been applied and brought to practical applicability by means of R language. Specifically, we tested five VIs for the Landsat 8–9 OLI/TIRS images and compared the results for the following indices: (1) Normalised Difference Vegetation Index (NDVI); (2) Normalised Difference Water Index (NDWI); (3) Infrared Percentage Vegetation Index (IPVI); (4) Optimised Soil-Adjusted Vegetation Index (OSAVI); and (5) Green Normalised Difference Vegetation Index (GNDVI).

The computational workflow of R libraries included the extraction of the image sensor DN values of pixels, computing the data range with min/max values, classifying the raster object and visualising the image using selected colour schemes. Within all of these methods, the data processing velocity can be estimated as higher compared to conventional GIS approaches, which offers additional possibilities for programming geospatial data analysis and image processing. As such, we demonstrated the usefulness of the R programming language for machine-based information extraction from satellite images. We also showed the effectiveness of the remote sensing data as a source of information derived from Earth observation satellites for regional studies focused on northeast Africa.

Image analysis in Earth sciences has made big progress due to the introduction of programming applications and scripts that significantly automate the routine of data processing. In the near future, we can expect the possibility of stream processing applied for large collections of satellite images as a series of data for operative monitoring. Thus, the further environmental applications of image processing could, for instance, include the monitoring of burned areas in savannah fires using satellite scenes processed straight after they are generated by sensors. Furthermore, the use of scripts for image processing opens the way for the geospatial analysis of diverse objects and structures of very high complexity that constitute the landscapes of the Earth. This includes the detailed diversification of land cover types, vegetation associations and plant communities that can be identified from images.

In monitoring the Earth’s landscapes, it is important to not only use topographic data, but also the environmental characteristics that reflect the complex interactions between the climate and hydrological parameters, as well as vegetation responses to external climatic effects. These can all be derived from satellite images such as Landsat 8–9 OLI/TIRS using advanced image processing methods, as demonstrated in this paper. Future research will be focused on developing further approaches using scripting algorithms that deal with satellite images to detect other parameters of land surface and vegetation coverage for environmental mapping, such as brightness and saturation indices, or evaluating other vegetation indices for detecting the salinity of soil in the arid lands of Africa. Such characteristics reflect the climate–vegetation interactions that can be used for operational environmental monitoring based on spaceborne data.

## Figures and Tables

**Table 1:** Satellite images used for computing the VIs: Landsat-8 OLI/TIRS collected from the USGS.

Date	Spacecraft	Landsat Product ID	Scene ID
20 December 2013	Lands. 8	LC08_L2SP_173049_20131220_20200912_02_T1	LC81730492013354LGN01
18 December 2013	Lands. 8	LC08_L2SP_173049_20181218_20200830_02_T1	LC81730492018352LGN00
21 December 2013	Lands. 9	LC09_L2SP_173049_20221221_20221224_02_T1	LC91730492022355LGN00

**Table 2:** Evaluation results of the VI values derived from the Landsat 8 OLI/TIRS images over the Khartoum region area (2013, 2018 and 2022).

VI	2013	2018	2022
$NDVI_{min}$	−0.2742168	−0.2817099	−0.2791084
$NDVI_{max}$	0.7246868	0.5796811	0.6044010
$GNDVI_{min}$	−0.2387020	−0.2830099	−0.2253696
$GNDVI_{max}$	0.6371941	0.5690726	0.7235179
$NDWI_{min}$	−0.4046338	−0.5656914	−0.5743572
$NDWI_{max}$	0.3880472	0.3764042	0.4152763
$OSAVI_{min}$	−0.2742153	−0.2817084	−0.2791067
$OSAVI_{max}$	0.7246833	0.5796785	0.6043976
$IPVI_{min}$	0.1376566	0.2101595	0.1977995
$IPVI_{max}$	0.6371084	0.6408550	0.6395542

## Data Availability

The source Landsat 8–9 OLI/TIRS images were downloaded from the EarthExplorer United States Geological Survey (USGS) data portal. URL: https://earthexplorer.usgs.gov/ (accessed on 13 February 2023).

## Abbreviations

The following abbreviations are used in this manuscript:

ENVI	ENvironment for Visualizing Images
ERDAS Imagine	Earth Resources Data Analysis System Imagine
GIS	Geographic Information System
GMT	Generic Mapping Tools
GNDVI	Green Normalised Difference Vegetation Index
IPVI	Infrared Percentage Vegetation Index
Landsat 8–9 OLI/TIRS	Landsat 8–9 Operational Land Imager (OLI) and Thermal Infrared (TIRS)
NDVI	Normalised Difference Vegetation Index
NDWI	Normalised Difference Water Index
OSAVI	Optimised Soil-Adjusted Vegetation Index
SAVI	Soil-Adjusted Vegetation Index
USGS	United States Geological Survey
UTM	Universal Transverse Mercator
VIs	Vegetation Indices
WGS84	World Geodetic System 1984

## Appendix A. Metadata on the Landsat 8–9 OLI/TIRS Images (USGS EarthExplorer)

**Table A1:** Metadata on the Landsat OLI/TIRS satellite images used in this study.

Dataset Attribute	Attributes for Image on 21 December 2018	Attributes for 18 December 2018	Attributes for 20 December 2018
Landsat Scene Identifier	LC91730492022355LGN01	LC81730492018352LGN00	LC81730492013354LGN01
Date Acquired	21 December 2018	18 December 2018	20 December 2018
Collection Category	T1	T1	T1
Collection Number	2	2	2
WRS Path	173	173	173
WRS Row	49	49	49
Target WRS Path	173	173	173
Target WRS Row	49	49	49
Nadir/Off Nadir	NADIR	NADIR	NADIR
Roll Angle	−0.001	0.000	0.000
Date Product Generated L2	17 March 2023	30 August 2020	12 September 2020
Date Product Generated L1	17 March 2023	30 August 2020	12 September 2020
Start Time	21 December 2022 08:09:26	18 December 2018 08:08:54.736348	20 December 2013 08:10:29.988456
Stop Time	21 December 2022 08:09:58	18 December 2018 08:09:26.506347	20 December 2013 08:11:01.758452
Station Identifier	LGN	LGN	LGN
Day/Night Indicator	DAY	DAY	DAY
Land Cloud Cover	0.00	0.00	0.00
Scene Cloud Cover L1	0.00	0.00	0.00
Ground Control Points Model	511	521	639
Ground Control Points Version	5	5	5
Geometric RMSE Model	7.263	6.387	4.378
Geometric RMSE Model X	5.055	4.612	2.951
Geometric RMSE Model Y	5.216	4.418	3.234
Processing Software Version	LPGS_16.2.0	LPGS_15.3.1c	LPGS_15.3.1c
Sun Elevation L0RA	44.22152569	44.38827439	44.40055924
Sun Azimuth L0RA	148.68774152	148.91482828	149.05309430
TIRS SSM Model	N/A	FINAL	ACTUAL
Data Type L2	OLI_TIRS_L2SP	OLI_TIRS_L2SP	OLI_TIRS_L2SP
Sensor Identifier	OLI_TIRS	OLI_TIRS	OLI_TIRS
Satellite	9	8	8
Product Map Projection L1	UTM	UTM	UTM
UTM Zone	36	36	36
Datum	WGS84	WGS84	WGS84
Ellipsoid	WGS84	WGS84	WGS84
Scene Center Lat DMS	15°54${}^{\prime }$03.06${}^{\prime \prime }$ N	15°54${}^{\prime }$03.56${}^{\prime \prime }$ N	15°54${}^{\prime }$03.42${}^{\prime \prime }$ N
Scene Center Long DMS	33°06${}^{\prime }$32.69${}^{\prime \prime }$ E	33°07${}^{\prime }$35.51${}^{\prime \prime }$ E	33°05${}^{\prime }$59.28${}^{\prime \prime }$ E
Corner Upper Left Lat DMS	16°56${}^{\prime }$34.84${}^{\prime \prime }$ N	16°56${}^{\prime }$44.88${}^{\prime \prime }$ N	16°56${}^{\prime }$44.41${}^{\prime \prime }$ N
Corner Upper Left Long DMS	32°02${}^{\prime }$24.68${}^{\prime \prime }$ E	32°03${}^{\prime }$25.49${}^{\prime \prime }$ E	32°01${}^{\prime }$44.08${}^{\prime \prime }$ E
Corner Upper Right Lat DMS	16°56${}^{\prime }$30.70${}^{\prime \prime }$ N	16°56${}^{\prime }$40.13${}^{\prime \prime }$ N	16°56${}^{\prime }$40.63${}^{\prime \prime }$ N
Corner Upper Right Long DMS	34°10${}^{\prime }$42.96${}^{\prime \prime }$ E	34°11${}^{\prime }$43.87${}^{\prime \prime }$ E	34°10${}^{\prime }$12.61${}^{\prime \prime }$ E
Corner Lower Left Lat DMS	14°50${}^{\prime }$38.76${}^{\prime \prime }$ N	14°50${}^{\prime }$39.01${}^{\prime \prime }$ N	14°50${}^{\prime }$38.58${}^{\prime \prime }$ N
Corner Lower Left Long DMS	32°03${}^{\prime }$00.32${}^{\prime \prime }$ E	32°04${}^{\prime }$00.55${}^{\prime \prime }$ E	32°02${}^{\prime }$20.18${}^{\prime \prime }$ E
Corner Lower Right Lat DMS	14°50${}^{\prime }$35.16${}^{\prime \prime }$ N	14°50${}^{\prime }$34.87${}^{\prime \prime }$ N	14°50${}^{\prime }$35.34${}^{\prime \prime }$ N
Corner Lower Right Long DMS	34°09${}^{\prime }$59.18${}^{\prime \prime }$ E	34°10${}^{\prime }$59.41${}^{\prime \prime }$ E	34°09${}^{\prime }$29.09${}^{\prime \prime }$ E
Scene Center Latitude	15.90085	15.90099	15.90095
Scene Center Longitude	33.10908	33.12653	33.09980
Corner Upper Left Latitude	16.94301	16.94580	16.94567
Corner Upper Left Longitude	32.04019	32.05708	32.02891
Corner Upper Right Latitude	16.94186	16.94448	16.94462
Corner Upper Right Longitude	34.17860	34.19552	34.17017
Corner Lower Left Latitude	14.84410	14.84417	14.84405
Corner Lower Left Longitude	32.05009	32.06682	32.03894
Corner Lower Right Latitude	14.84310	14.84302	14.84315
Corner Lower Right Longitude	34.16644	34.18317	34.15808

## Appendix B. R Scripts Used for Computing and Plotting the Vegetation Indices

### Appendix B.1. R Code for Computing the NDVI

**Listing A1.** R code for computing the NDVI (here: a case from 2022).

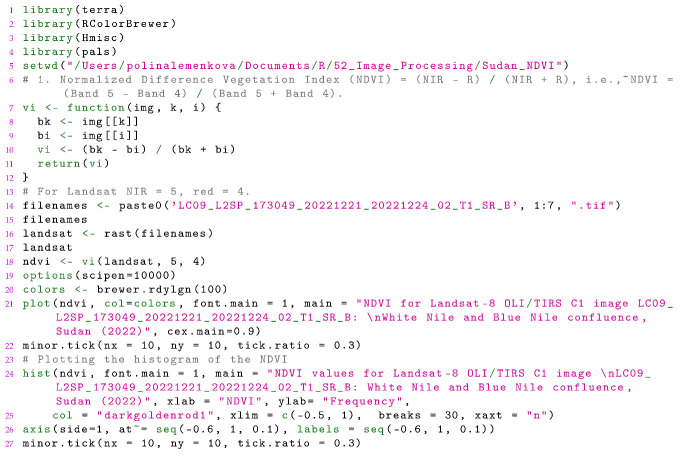

### Appendix B.2. R Code for Computing the GNDVI

**Listing A2.** R code for computing the GNDVI (here: a case from 2013).

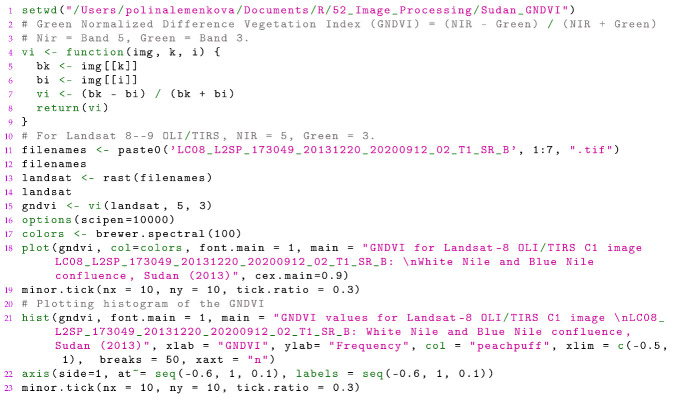

### Appendix B.3. R Code for Computing the NDWI

**Listing A3.** R code for computing the NDWI (here: a case from 2018).

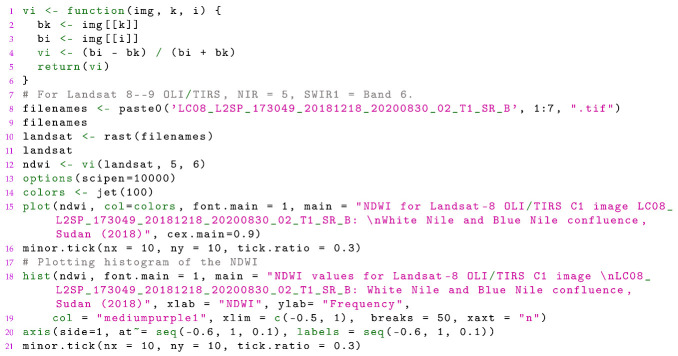

### Appendix B.4. R Code for Computing the OSAVI

**Listing A4.** R code for computing the OSAVI (here: a case from 2022).

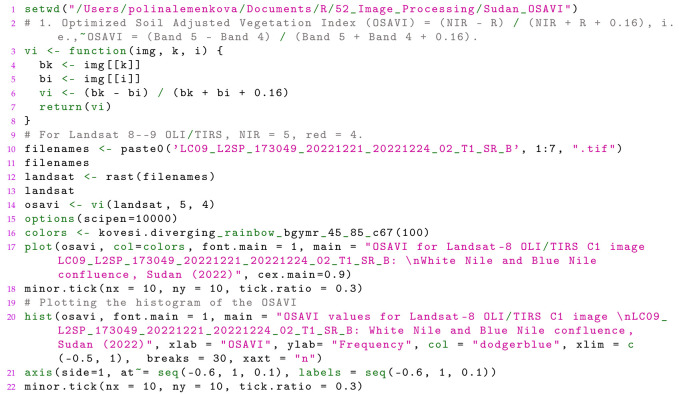

### Appendix B.5. R code for computing the IPVI

**Listing A5.** R code for computing the IPVI (here: a case from 2013).
